# Epigenetic Regulation of Angiogenesis in Peripheral Artery Disease

**DOI:** 10.14797/mdcvj.1294

**Published:** 2023-11-16

**Authors:** Naseeb Kaur Malhi, Kevin W. Southerland, Li Lai, Zhen Bouman Chen

**Affiliations:** 1 Beckman Research Institute City of Hope, Duarte, California, US; 2 Duke University Medical Center, Durham, North Carolina, US; 3 Houston Methodist Research Institute, Houston, Texas, US

**Keywords:** epigenetics, peripheral arterial disease (PAD), hindlimb ischemia (HLI), angiogenesis, long non-coding RNAs (lncRNAs), fibroblast-endothelial transdifferentiation

## Abstract

Peripheral arterial disease (PAD) represents a global health concern with a rising prevalence attributed to factors such as obesity, diabetes, aging, and smoking. Among patients with PAD, chronic limb-threatening ischemia (CLTI) is the most severe manifestation, associated with substantial morbidity and mortality. While revascularization remains the primary therapy for CLTI, not all patients are candidates for such interventions, highlighting the need for alternative approaches. Impaired angiogenesis, the growth of new blood vessels, is a central feature of PAD, and despite decades of research, effective clinical treatments remain elusive. Epigenetics, the study of heritable changes in gene expression, has gained prominence in understanding PAD pathogenesis. Here, we explore the role of epigenetic regulation in angiogenesis within the context of PAD, with a focus on long non-coding RNAs and fibroblast-endothelial cell transdifferentiation. Additionally, we discuss the interplay between metabolic control and epigenetic regulation, providing insights into potential novel therapeutic avenues for improving PAD treatments. This review aims to offer a concise update on the application of epigenetics in angiogenesis and PAD research, inspiring further investigations in this promising field.

## Introduction

Peripheral arterial disease (PAD), which is defined as arterial occlusive lesions in the lower extremity, is a major cause of morbidity and mortality. Currently, 200 million individuals worldwide are affected by PAD,^1^ with the number predicted to rise as a result of increased detrimental environmental factors such as obesity, diabetes, aging, and smoking.^2^ Approximately 10% of PAD patients present with the most severe clinical manifestation, termed chronic limb-threatening ischemia (CLTI).^3^ CLTI, which presents as rest pain, nonhealing wounds, or gangrene is a devastating pathology with a 1-year limb loss rate of 25% and a 5-year mortality of 50%.^4,5^ The primary therapy for CLTI patients is surgical or endovascular revascularization.^6^ Despite vast improvements in revascularization strategies, amputation rates remain high. In fact, up to 20% of CLTI patients are not candidates for revascularization attempts based on anatomic constraints.^7^ Hence, there is a great need for molecular therapies aimed at improving perfusion to the ischemic limb.

Among the many damaging functional aspects contributing to PAD, impaired angiogenesis is one of the most prominent and intensively investigated. Angiogenesis is defined as the growth and proliferation of blood vessels from existing vasculature. In the context of PAD, the hypoxic and ischemic environment triggers the release of growth factors that act upon the endothelial cells (ECs), causing them to proliferate, migrate, and elongate to expand the microvasculature. This angiogenic process can be therapeutically enhanced by growth factors such as vascular endothelial growth factor (VEGF). Therapeutic angiogenesis has been investigated for over two decades for its potential clinical use to treat PAD.^8,9^ However, none of these therapies have translated to clinical success yet.

Epigenetics investigates the heritable changes in gene expression that occur without alterations to the underlying DNA sequence. The aforementioned environmental factors currently plaguing the western world, such as obesity and diabetes, are implicated in promoting epigenetic changes. Some of the key molecular processes under investigation by epigenetics include DNA methylation (DNAme), histone modifications, long non-coding RNAs (lncRNAs), and chromatin remodeling. During the induction of angiogenesis, ECs can undergo epigenetic changes, in which the quiescent ECs become activated and mobilized. In the context of PAD, epigenetic modifications have been shown to play a pivotal role in the development and progression of this vascular disorder through influencing the expression of genes involved in not only angiogenesis but also other contributing factors such as inflammation, oxidative stress, and vascular remodeling. Understanding how epigenetic changes contribute to the onset and progression of PAD not only deepens our comprehension of its underlying mechanisms but also offers potential avenues for therapeutic interventions and personalized treatment strategies.

In this review, we will focus on epigenetic regulation, angiogenesis, and PAD. We will provide an overview of epigenetic regulation and its role in angiogenesis in the context of PAD. Specifically, we will emphasize the role of lncRNAs, which are showing potential as novel targets to achieve therapeutic angiogenesis. We also will discuss fibroblast-EC transdifferentiation, an alternative source of angiogenesis and an epigenetic cell-reprogramming process that may be leveraged to develop therapeutics for PAD. Through discussion of fibroblast-EC transdifferentiation, we will touch upon the connection between metabolic control and epigenetic regulation, two key aspects of cell biology. This review is not meant to be exhaustive nor comprehensive. Instead, we intend to provide readers with a brief update on the application of epigenetics in angiogenesis and PAD research and spur interest in future efforts in leveraging epigenetics to improve PAD treatments.

## Overview of Epigenetic Regulation

In a broad sense, epigenetics bridges between genotype and phenotype.^9^ For DNAme and histone modifications, there are numerous writers, readers, and erasers—for example, enzymes that mediate the addition, processing, and removal of the modifications. Interested readers are referred to extensive reviews on these topics.^10,11,12,13^

### DNA Methylation

The first identified epigenetic regulation, DNAme, involves the addition of methyl groups to specific cytosine bases in DNA, typically at CpG dinucleotides, which are enriched in the promoter regions. In most cases, DNAme can repress gene transcription and is often associated with gene silencing. DNAme has been extensively studied in cancer, with the promoter regions of many tumor suppressor genes hypermethylated. Homocysteine, a circulating marker of PAD, is known to mediate DNAme.^14^ One prominent drug used to target DNAme is 5-azacytidine, often referred to as 5-aza, a demethylating agent applied to some cancers, including acute myeloid leukemia and myelodysplastic syndrome. Importantly, 5-aza has been shown to ameliorate atherosclerosis through suppressing macrophage inflammation in mice^15^; however, the importance of DNAme directly in PAD has been little studied.

### Histone Modifications

Histone modifications encompass over a hundred chemical alterations made to histone proteins, such as acetylation, methylation, phosphorylation, and ubiquitination, to name a few well-studied ones. These histone modifications play a crucial role in regulating gene expression by either loosening or tightening the chromatin structure, thereby controlling the accessibility of genes to transcriptional machinery. Different combinations of these modifications create a “histone code” that governs various cellular processes, and the dysregulation of histone modifications has been implicated in numerous diseases.

Like DNAme, our knowledge of histone modifications has come primarily from cancer research. One of the best-known examples of histone modification and its relevance to disease is the role of histone acetylation in cancer. In many cancer types, there is an aberrant increase in histone deacetylase (HDAC) activity, leading to the removal of acetyl groups from histone proteins. This deacetylation results in a more condensed chromatin structure, which can silence tumor suppressor genes and promote oncogene expression. HDAC inhibitors have been developed to treat lymphoma and have shown promise in treating other cancer types. A histone deacetylase inhibitor, MCT-1, has shown some promise in rat models of another type of PAD, abdominal aortic aneurysm.^16^ However, similar to DNAme, histone modifications in PAD also have been rarely studied.

### Long Non-coding RNAs

**Figure 1:**
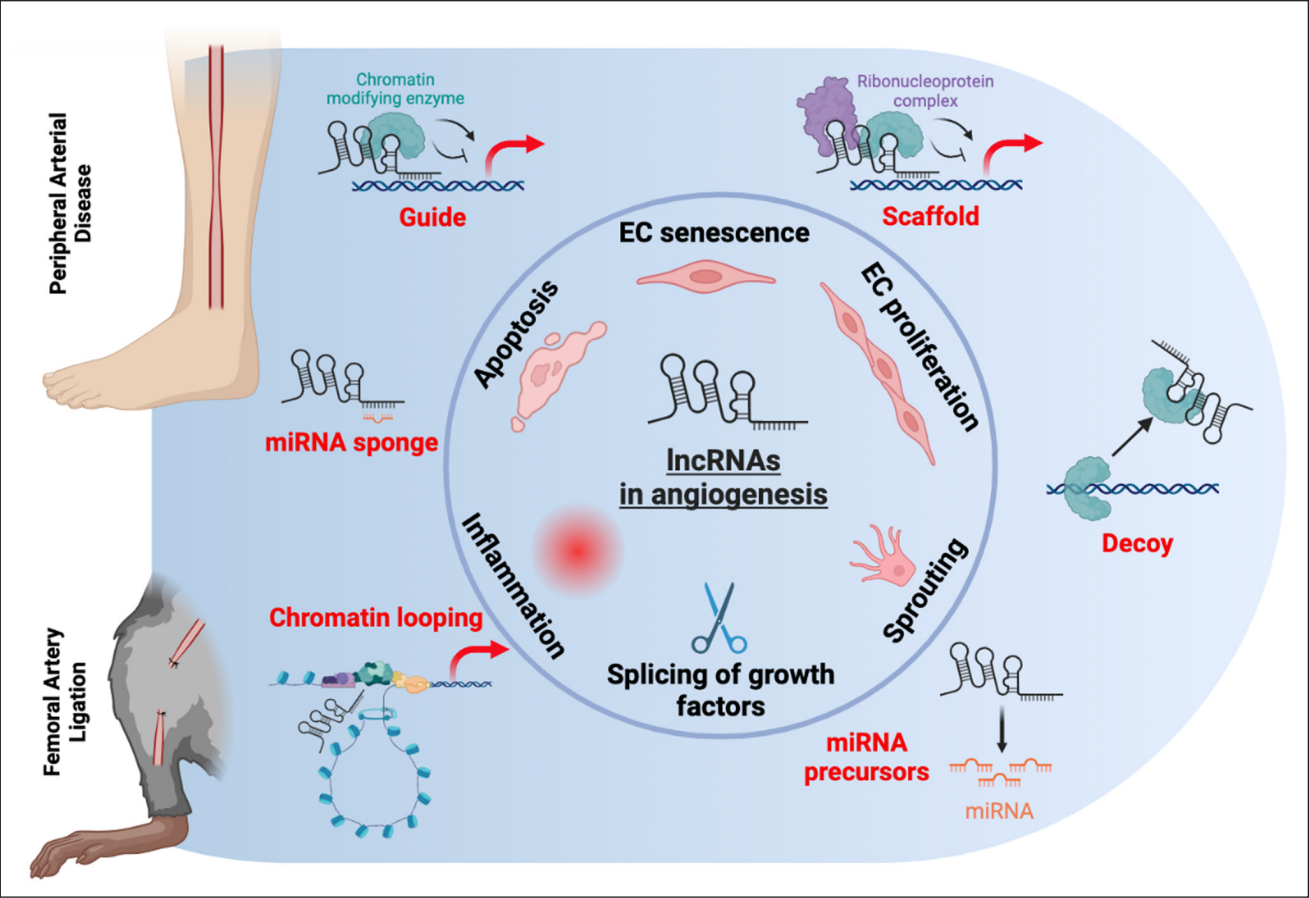
Potential modes of action of lncRNA in angiogenesis in peripheral arterial disease. LncRNAs are regulators of angiogenic pathways in endothelial cells, including their senescence and activation, proliferation, sprouting, and apoptosis. They also may modulate the expression of growth factors via splicing. In addition, they are involved in inflammation, a key driver of angiogenesis. The potential mechanisms through which they deploy their action are guiding, scaffolding, or decoying chromatin modifying enzymes. They also may act as miRNA sponges or precursors and are involved in chromatin structure via looping. lncRNAs: long non-coding RNAs; miRNA: microRNA

Long non-coding RNAs (lncRNAs) are generally defined as RNA transcripts over 200 nucleotide in length that do not code for proteins but instead function as regulatory molecules. Depending on their subcellular localization, lncRNAs can be nuclear, chromatin-bound or cytoplasmic (Figure 1). Those involved in epigenetic regulation are typically nuclear localized and chromatin-associated, which we refer to as chromatin-associated lncRNAs (ca-lncRNAs). Some of these ca-lncRNAs can interact with chromatin-modifying complexes, such as histone methyltransferases or chromatin remodelers, to influence the structure and accessibility of chromatin. Other lncRNAs can act as decoys, scaffolds, or guides for transcription factors, RNA polymerases, and other regulatory proteins, helping to fine-tune gene expression. These can either enhance or inhibit transcription depending on the specific lncRNA and its interactions. LncRNAs can also mediate genomic imprinting, a process where specific genes are expressed based on their parental origin. For example, the lncRNA XIST is essential for X-chromosome inactivation in females.^17,18^ Additionally, lncRNAs can help maintain epigenetic memory across cell divisions by recruiting chromatin-modifying enzymes to specific genomic loci, ensuring the stable transmission of epigenetic marks.

A number of excellent reviews summarizing the mode-of-action of lncRNAs and their implications in various diseases are available for interested readers.^19,20,21,22^ Pertaining to PAD, a recent review has summarized the role of ncRNAs, including microRNAs and lncRNAs, as functional mediators or biomarkers in the pathophysiology of CLTI.^23^ In the next section, we will focus on lncRNAs as epigenetic regulations in angiogenesis in the context of PAD, and we will provide several examples how these lncRNAs can be involved in the pathogenesis of PAD. A better understanding of these interesting regulators may aid in the development of improved therapeutics to treat PAD.

## Role of lncRNAs in Angiogenesis in PAD

To date, dozens of lncRNAs have been reported to play a regulatory role in angiogenesis (Table 1), and the list is still increasing. Highlighted here are several that have been implicated in PAD due to its regulation of angiogenesis.

**Table 1:** Reported long non-coding RNAs with functional relevance in peripheral arterial disease.^24,25,26,27,28,29,30,32,33,36,38,39,40,41,42,44,45,46,47,48,49,50,51,52,53,54,55,56,57,58,59,60,61,62,63^ ECs: endothelial cells; KO: knockout; HLI: hindlimb ischemia; miR: microRNA; eNOS: endothelial nitric oxide synthase; KDR: kinase insert domain receptor; PBMCs: peripheral blood mononuclear cells; PAD: peripheral artery disease; VEGF: vascular endothelial growth factor; SNP: single nucleotide polymorphisms; VSMCs: vascular smooth muscle cells; BRG1: Brahma-related gene 1


LNCRNA	CELL SPECIFICITY	RELATIONSHIP TO PAD	PROPOSED MECHANISM

**MALAT1** ^24,25,26,27,28^	Multiple cell types including ECs	Reduced blood flow recovery in KO model of HLI, involved in EC proliferation	VEGFR2

**MEG3** ^29,30,32,33^	Multiple cell types including ECs and fibroblasts	Improved blood flow recovery in KO model of HLI, expressed in senescent ECs and reduced during EC sprouting	Notch signaling, miR-21

**ANRIL** ^36,38,39,40,41,42^	Multiple cell types including ECs, myocytes and fibroblasts	cdkn2b-deficient mouse under HLI showed reduced blood flow recovery and higher digital amputation	TGF-βR1/Smad pathway, eNOS

**SNHG12** ^44,45^	Multiple cell types including ECs, immune cells and fibroblasts	In KO mice, impaired angiogenic response to HLI, more pronounced in diabetes	Wnt, Notch, and angiopoietin signaling pathways

**LEENE** ^46,47,48,49^	EC enriched	Impaired perfusion recovery in HLI of KO mice, restored with LEENE expression	KDR, eNOS

**STEEL** ^50,51,52^	Endothelial enriched	Stimulates formation and maturation of vascular flow networks; decreased expression in disturbed flow	KD reduces expression of shear stress related genes KLF2, eNOS; feedback loop with KLF2

**H19** ^52,53,54^	Multiple cell types including ECs, myoblasts and PBMCs	Increased in PAD mouse model after 14 days of ischemia, involved in myogenesis, reduced capillary density EC specific-KO model of HLI	STAT3 signaling; IL-6; VEGF

**MIR22HG** ^52,54^	Multiple cell types including ECs	Elevated in hypoxic ECs and HLI model	Unknown

**LINC00607** ^55,56,57^	ECs and VSMCs	Associated SNP found predisposing patients to PAD	c-Myc

**HIF1A-AS1** ^58,59^	VSMCs and ECs	Hypoxia regulated element	BRG1

**SENCR** ^52,60^	Highest in ECs but also expressed in VSMCs	Expression is reduced in human critical limb ischemia patients	CX1CL3, CCL5, CEACAM1

**FENDRR** ^61^	ECs and VSMCs	Decreased in hypoxia	DRP1 DNA methylation, p53

**HIF1A-AS2** ^62,63^	VSMCs and ECs	Hypoxia regulated element	USF1 to elevate ATF2


### MALAT1

Metastasis-Associated Lung Adenocarcinoma Transcript 1 (MALAT1) is one of the most highly studied and conserved lncRNA. It has been associated with numerous disorders, including vascular diseases, and is one of the most abundant lncRNA transcripts in all cells including ECs. Importantly, it was found to promote EC proliferation but reduce EC migration and sprouting. Inhibition of MALAT1 in mice reduced blood flow recovery in femoral artery ligation-induced hindlimb ischemia (HLI), a mouse model of PAD.^24,25^ A global knockout of MALAT1 induced similar results, hypothesized to be mediated through vascular endothelial growth factor receptor 2 binding.^26^ Moreover, reduced MALAT1 levels were found in human atherosclerotic lesions of symptomatic patients, associated with a poor prognosis. In macrophages, MALAT1 was found to enhance the biological functions of high glucose-impaired macrophages, leading to improved phagocytosis, a shift towards a pro-healing phenotype, reduced apoptosis, and thus ultimately promoting the healing of diabetic wounds.^27^ However, another study concluded MALAT1 overexpression increased inflammation and EC dysfunction in diabetes.^28^ Thus, while it is clear that MALAT1 is involved in EC function and vascular repair, the effect of its modulation may be more context-dependent.

### MEG3

Maternally expressed gene 3 (MEG3), transcribed from a maternally imprinted gene, is another highly expressed lncRNA in ECs. Increased in senescent ECs, its inhibition led to improved EC sprouting and migration in vitro and restoration of blood perfusion in mice with HLI.^29^ This effect is thought to be mediated in part by the Notch signaling pathway and by inducing microRNA-21,^30^ a positive regulator of angiogenesis.^31^ In the brains of MEG3-knockout mice, there was significantly higher cortical microvessel density compared with controls, coupled with an increased expression of angiogenesis-related genes such as VEGF and Notch1.^32^ Of note, the DNAme in 14q32 locus, where MEG3 is transcribed from, has been associated with type 2 diabetes, atherosclerosis, and PAD.^33,34,35^

### ANRIL

Antisense non-coding RNA in the INK4 Locus (ANRIL) is transcribed from human chromosome 9p21,^36^ a susceptibility locus of coronary artery disease now considered the most robust genetic marker of atherosclerotic cardiovascular disease, including PAD.^37^ Intriguingly, linear isoforms of ANRIL are associated with increased atherosclerosis susceptibility and an elevated risk of atherosclerotic plaques. In contrast, circular isoforms of ANRIL offer protection against atherosclerotic plaques and mitigate the risk of atherosclerosis.^38^ The different functions of distinct isoforms of ANRIL may be related to seemingly inconsistent findings regarding the role of ANRIL in EC function. For instance, in the context of chronic kidney disease, ANRIL expression was found to increase with uremia toxin, which also increased expression of inflammatory cytokines in ECs. Inhibition of ANRIL was shown to alleviate endothelial dysfunction in mouse models of chronic kidney disease.^39^

Consistently, another study showed that knockdown of ANRIL reduced apoptosis and inflammation and increased proliferation and tube formation of human umbilical vein endothelial cells (HUVECs), which was suggested to be mediated by the TGF-βR1/Smad pathway.^40^ A more recent study found that ANRIL regulates the inflammatory response of HUVECs through regulating alternative splicing.^41^ However, inhibition of ANRIL has also been shown to reduce endothelial nitric oxide synthase (eNOS) protein levels and nitric oxide (NO) release from HUVECs by other studies. In fact, ANRIL overexpression was shown to increase eNOS expression and promoted post-ischemic angiogenesis while improving cardiac function in mice following myocardial ischemia.^42^ ANRIL is not conserved in mice, but deficiency of *CDKN2B*, a gene found in the 9p21 locus, showed increased tissue necrosis and reduced blood flow in mice undergoing HLI.^43^

### SNHG12

The evolutionary conserved small nucleolar host gene 12 (SNHG12) was found to be decreased in aortic ECs in atherosclerotic lesions.^44^ The same group then investigated its role in angiogenesis and PAD and found that SNHG12 was decreased in ischemic ECs and increased with perfusion recovery. In mice with SNHG12 inhibition, there was an impaired angiogenic response, which was more pronounced in diabetic (*db/db*) mice, suggesting a positive effect of SNHG12 in angiogenesis and neovascularization. Of note, despite relatively similar knockdown of SNHG12 in both the EC and non-EC fractions, RNA-seq analysis showed different enriched pathways encompassing inflammation and angiogenesis between the two fractions. This points to a need to evaluate the tissue/cell type-specific role of lncRNAs in ischemic response.^45^

### LEENE

LncRNA that enhances eNOS expression (LEENE) is a novel regulator in EC biology, angiogenesis, and ischemic response. It is encoded by *LINC00520*, an enhancer region in human ECs. It was found that both the chromatin accessibility and the transcriptional activity of *LINC00520* was enhanced by flow, particularly by shear stress.^46,47^ LEENE expression was also increased by hypoxia in ECs but exhibited a decline under diabetic conditions, evident in cultured ECs, mouse hindlimb muscles, and human arteries. Inhibition of LEENE within human microvascular ECs led to a reduction in their angiogenic capacity, accompanied by disruptions in the normal angiogenic gene program. In a murine model of diabetes, mice lacking the mouse homolog of human LEENE displayed compromised angiogenesis and impaired perfusion after HLI, an experimental model of PAD. Notably, the introduction of human LEENE overexpression successfully rectified the impaired ischemic response in *leene*-knockout mice, both at the level of tissue function and in single-cell transcriptomic analyses. The potential augmentation of LEENE functioned to reinstate angiogenesis, thereby aiding tissue repair and regeneration, may be a promising strategy in addressing PAD.^48,49^

## Mesenchymal-EC Transdifferentiation and Metabolic Regulation of Epigenetics

While promoting the angiogenic capacity of existing ECs is a direct approach to enhance angiogenesis and improve blood flow perfusion in ischemic tissues, another promising approach is to enhance angiogenic transdifferentiation from tissue fibroblasts into ECs.^64^ The contribution of such transdifferentiation to vascular regeneration has been demonstrated in the murine HLI model using the fibroblast lineage tracing approach and single cell fate mapping.^65,66^ Through the transdifferentiation process, fibroblasts acquire the gene expression profile and functional characteristics of microvascular ECs.^64^ During this event, thousands of genes are up- or down-regulated, accompanied by a remarkable epigenetic reprogramming.^67,68,69^ As a result, the metabolic activity of the transdifferentiated cells becomes drastically different from the original state, signified by a glycolytic switch.

While such metabolic alterations may seem to be passive adaptations as the mesenchymal cells get converted to endothelial lineage, recent studies have pointed to an active role of metabolic modulation on the epigenetic reprogramming of the fibroblasts.^67^ A glycolytic switch was found to occur before the expression of EC markers in transdifferentiating fibroblasts. Inhibiting glycolysis impaired the generation of transdifferentiated ECs, whereas promoting glycolysis enhanced the transdifferentiation. Furthermore, during this process, nuclear ATP-citrate lyase (ACL/ACLY) was increased, along with citrate, which is converted to acetyl-CoA by ACL. Knockdown of ACL/ACLY attenuated both glycolytic switch and the consequent transdifferentiation. These findings suggest a novel role of ACL in angiogenic transdifferentiation and also highlight the link between metabolism and epigenetic modulation in transdifferentiation.

**Figure 2:**
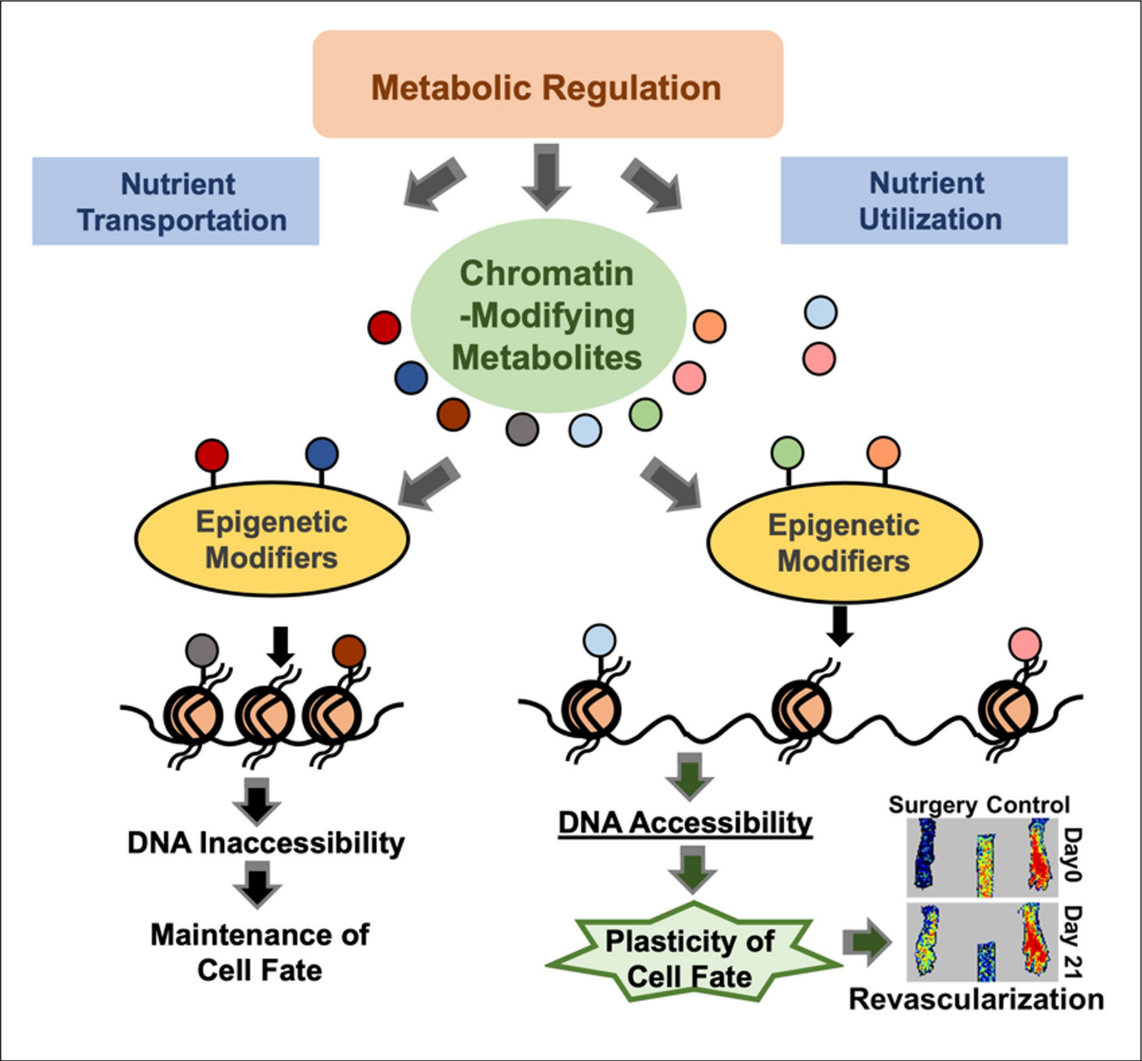
Metabolic regulation on epigenetics. The chromatin-modifying metabolites, regulated by their rate-limiting enzymes and the availability of their precursors, can bind to histone tails or epigenetic modifiers to affect chromatin accessibility, which further modulates the cell fate transition.

Acetyl-CoA is the substrate for histone acetylation, which increases the accessibility of chromatin to transcriptional activation.^70,71,72,73^ Thus, the increase in the nuclear ACL can increase the nuclear pool of acetyl-CoA, increasing histone acetylation and promoting the chromatin accessibility necessary for the transdifferentiation. Aside from acetyl-CoA,^74,75^ many other metabolites, such as S-adenosylmethionine, succinate,^76^ lactate,^77^ α-ketoglutarate, and uridine diphosphate N-acetylglucosamine, are also essential building blocks, cofactors, and substrates for epigenetic processes.^78^ Many of these metabolites can couple with epigenetic modifiers to influence chromatin states to maintain or alter cell fate (Figure 2). Therefore, better understanding of the metabolic-epigenetic crosstalk can provide valuable insights and therapeutic strategies to promote angiogenic transdifferentiation and tissue regeneration.

## Conclusion and Perspective

The emerging field of epigenetic regulation in angiogenesis holds immense promise in shedding light on the intricate mechanisms underlying PAD. The dynamic interplay between epigenetic modifications and the angiogenic processes within the vascular system has unveiled new avenues for understanding the pathogenesis of PAD and exploring innovative therapeutic strategies. For example, diabetes is a prominent risk factor for PAD, and the hyperglycemic condition is well known to induce heritable epigenetic changes. Elucidating how diabetic conditions may modulate epigenome supporting the angiogenic function of ECs may facilitate the design of more effective therapies to intervene in diabetic PAD. Similarly, understanding how epigenetic variations contribute to PAD heterogeneity may enable ways to stratify patients into distinct subgroups, each with tailored treatment approaches. In addition, understanding the mechanisms underlying the metabolic-epigenetic control of fibroblast-EC transdifferentiation may make it possible to augment the cell plasticity and maximize the transdifferentiation potential, which is desirable in regenerative medicine for PAD. While many drugs targeting the epigenome are being tested for treatment of cancer and other cardiovascular diseases, future research is warranted to determine the promise of using these epigenetic drugs in the treatment of PAD.

As we deepen our comprehension of the epigenetic regulatory mechanisms, we are poised to develop targeted interventions that may mitigate the progression of PAD and restore vascular function to improve the quality of life for those affected by this debilitating condition. This intersection of epigenetics and angiogenesis in PAD research represents a promising frontier in the quest for more effective treatments and enhanced patient outcomes.

## Key Points

- Epigenetic regulatory mechanisms are important modulators of angiogenesis that may be harnessed to treat peripheral arterial disease.
- Histone modifications and DNA methylation regulate the epigenome to effect endothelial angiogenic capacity but require further study in peripheral arterial disease.
- Long non-coding RNAs are involved in most, if not all, steps of endothelial angiogenesis, from expression of growth factors to sprouting. Targeting these transcripts has recently shown therapeutic potential.
- Chromatin binding metabolites can alter the epigenome, to either maintain or alter cell fate, such as in fibroblast-EC transdifferentiation.
